# Data on the stability of Prx1-associated snRNAs and mRNAs

**DOI:** 10.1016/j.dib.2019.104309

**Published:** 2019-07-25

**Authors:** Eun-Kyung Kim, Sung-Min Ahn, Ho Hee Jang

**Affiliations:** a Department of Biochemistry, College of Medicine, Gachon University, Incheon, 21999, Republic of Korea; b Department of Genome Medicine and Science, College of Medicine, Gachon University, Incheon, 21936, Republic of Korea; c Division of Medical Oncology, Department of Internal Medicine, Gachon University Gil Medical Center, Incheon, 21565, Republic of Korea; d Department of Health Sciences and Technology, Graduate School of Medicine, Gachon University, Incheon, 21999, Republic of Korea

**Keywords:** Peroxiredoxin1, Small nuclear RNA, RNA stability, Protein-RNA interaction

## Abstract

This data set is related to the research article entitled “Peroxiredoxin 1 post-transcriptionally regulates snoRNA expression” (Kim et al., 2019). It demonstrates that peroxiredoxin 1 (Prx1) increases the stability of Prx1-associated small nuclear RNAs (snRNAs) and mRNAs. We overexpressed Prx1 in SNU484 and HeLa cells, which were then treated with Actinomycin D (ActD) to inhibit transcription. After that, we measured the levels of Prx1-associated snRNAs and mRNAs using qPCR analysis.

**Table:** Specifications Table

Subject	Biochemistry, Genetics and Molecular Biology (General)
Specific subject area	Gene expression; Post-transcriptional regulation
Type of data	Figure
How data were acquired	Quantitative RT-PCR analysis using ABI 7900HT Fast Real-Time PCR System (Applied Biosystems).
Data format	Analysed
Parameters for data collection	Cells were transfected with empty vector or Myc-Prx1 and cultured in serum starvation for 72 h. Cells were then treated with 5 μg/ml ActD for 90 min in SNU484 cells and 180 min in HeLa cells after replacing the complete culture medium.
Description of data collection	Total RNA was extract with TRIzol reagent and treated with DNase I. The cDNA was synthesized from 1 μg of total RNA by reverse-transcription PCR with a PrimeScript 1st Strand cDNA Synthesis Kit (Takara). The snRNA and mRNA levels were quantified using real-time PCR.
Data source location	Department of Biochemistry, College of Medicine, Gachon University, Incheon 21999, Republic of Korea
Data accessibility	Data is available with this article. The raw data file is provided in Supplementary data.
Related research article	Eun-Kyung Kim, Sun Young Lee, Yosup Kim, Sung-Min Ahn, Ho Hee Jang. Peroxiredoxin 1 post-transcriptionally regulates snoRNA expression. Free Radic. Biol. Med. 141 (2019) 1–9 [1].

**Table:** 

**Value of the data** • It provides an extra insight that Prx1 can bind to and increase the RNA stability of snRNAs and mRNAs. • Scientists investigating the biological consequences of protein-RNA interactions, especially, in the context of oxidative stress • In the related research article, we only focused on snoRNAs for various reasons, but the scope can be vastly expanded to snRNA and mRNA targets of Prx1.

## 1 Data

**Fig. 1:**
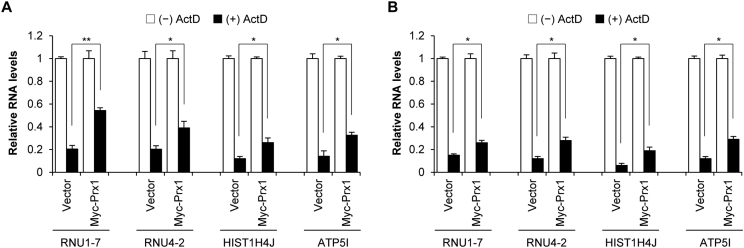
Stability of snRNAs (RNU1-7 and RNU4-2) and mRNAs (HIST1H4J and ATP5I) in Prx1-overexpressing cells. SNU484 cells or HeLa cells were transfected with either the empty control vector or the Prx1-containing vector. SNU484 cells were treated with ActD (5 μg/ml) for 90 min (A) and HeLa cells were for 180 min (B). RNA levels were measured with qPCR with cyclophilin as the normalized control of mRNA and U6 as the normalized control of snRNA. The data presented are the mean ± SD for three independent experiments (*P < 0.05, **P < 0.01).

We measured the levels of snRNA (RNU1-7 and RNU4-2) and mRNA (HIST1H4J and ATP5I) after treating cells with ActD to observe the changes in Prx1-associated snRNAs and mRNAs at the post-transcriptional level. SNU484 and HeLa cells were transfected with either the empty control vector or the Prx1-containing vector. We treated transfected SNU484 and HeLa cells with ActD for 90 min and 180 min, respectively, and measured the levels of snRNAs and mRNAs remaining before and after ActD treatment for comparison (Fig. 1 and Supplementary data).

## 2 Experimental design, materials, and methods

### 2.1 RNA stability assay

SNU484 and HeLa cells were maintained in RPMI and DMEM, respectively, containing 10% fetal bovine serum with 100 unit/ml penicillin/streptomycin at 37 °C in 5% CO_2_ atmosphere. Transfection were performed with pCS4 vector or pCS4-Myc-Prx1 plasmid using Lipofectamine 2000 according to the manufacturer's protocol (Invitrogen). The cells were synchronized at G0/G1 stage by serum starvation for 72 h and then exposed to 5 μg/ml ActD for indicated times with complete growth medium [2]. Total RNA was extract with TRIzol reagent at the indicated time points following the addition of ActD to block the synthesis of new transcripts. The cDNA was synthesized from 1 μg of total RNA by reverse-transcription PCR with a PrimeScript 1st Strand cDNA Synthesis Kit (Takara). Quantitative real-time PCR using SYBR Premix Ex Taq II was performed to compare the rate of RNA decay in the vector-transfected and Prx1-transfected cells. The human cyclophilin was used as control for mRNA normalization and U6 was used as control for snRNA normalization. The RNA levels were expressed relative to before ActD treatment. Primers used for this analysis are follows: RNU1-7, forward 5′ TGATCACGAAGGTGGTTTTCC 3′ and reverse 5′ GCACATCCGGAGTGCAATC 3’; RNU4-2, forward 5′ GCGCGATTATTGCTAATTGAAAA 3′ and reverse 5′ GCCAATGCCGACTATATTTCAAG 3’; HIST1H4J, forward 5′ ATGTCTGGCCGCGGCAAAGGC 3′ and reverse 5′ GCCGGCTTGGTGATGCCCTGG 3’; ATP5I, forward 5′ ATGGTGCCACCGGTGCAGGT 3′ and reverse 5′ TTAGGTAATTGTAGCGCGTGGC 3’; U6, forward 5′ GCTTCGGCAGCACATATACTAAAAT 3′ and reverse 5′ CGCTTCACGAATTTGCGTGTCAT 3’; Cyclophilin, forward 5′ TGCACAGACGGTCACTCAAA 3′ and reverse 5′ TGCCATCGCCAAGGAGTAG 3’.

### 2.2 Statistical analysis

All experiments were performed at least three times and data were presented as mean values ± SD. Statistical analysis was performed using one-way ANOVA. Comparisons between two groups were analysed using Student's t-test. Differences between data groups were considered significant at *P* < 0.05.
